# Recent advances in congenital heart disease genomics

**DOI:** 10.12688/f1000research.10113.1

**Published:** 2017-06-12

**Authors:** Anna Wilsdon, Alejandro Sifrim, Marc-Phillip Hitz, Matthew Hurles, J. David Brook

**Affiliations:** 1 School of Life Sciences, University of Nottingham, University Park, Nottingham, NG7 2RD, UK; 2 Wellcome Trust Sanger Institute, Hinxton, Cambridge, CB10 1SA, UK

**Keywords:** congenital heart disease, genomics, de novo mutations

## Abstract

Congenital heart disease is the most common congenital abnormality, and advances in medical care mean that this population of individuals is surviving for longer than ever before. It represents a significant healthcare challenge, as many patients require life-long care and individuals may ask about the likelihood of their children being affected. Whilst a number of genes have been identified previously from investigation of families with Mendelian inheritance patterns, sequencing the DNA from large cohorts of individuals with congenital heart disease is now providing fresh insights into the genetics of these conditions. This research has enabled novel gene discovery and uncovered the different genetic mechanisms underlying both isolated congenital heart disease and that which occurs in association with other medical problems. This article discusses the most recent advances in this field and the implications for patient care. In addition, we consider the challenges facing researchers in this field and emphasise the need for close working relationships between clinicians and researchers.

Congenital heart disease (CHD) is a structural abnormality in the heart that is present at birth. This can range from a simple “hole in the heart” (septal defect) that might not need any treatment to more severe abnormalities that require surgery soon after birth or within the first year of life. It affects around nine in every 1,000 babies born in the UK. Advances in surgical and medical treatments mean that individuals with CHD are surviving for longer and represent an expanding population with specific health needs. Many require life-long care because of the risk of developing primary and secondary complications of the heart valves, as well as arrhythmias, heart failure, and stroke ^1– 4^. They may also ask about the chance of any children they have being similarly affected.

Despite the fact that CHD usually occurs as a “one off” in a family, for a long while we have known that there is a genetic component, and more than 50 genes have been implicated so far ^5^. *De novo* mutations leading to reduced reproductive fitness were suspected owing to the sporadic nature of CHD. At the moment, we can identify a genetic cause in around 10–20% of people with CHD; this is much more likely in those who have CHD in combination with other medical issues, including neurodevelopmental disorders. Studies have already begun to look at the potential gains of genetic evaluation in certain clinical populations including neurodevelopmental clinics and cardiology units ^6, 7^.

In this article, we will discuss the recent advances in genomics in CHD and the challenges researchers are now facing. We will also consider how findings can be translated into improved clinical care for patients.

Traditionally, the discovery of novel human disease genes has employed analysis of pedigrees with a Mendelian family history of CHD and subsequent targeted testing in others with the same phenotype. Pitfalls of this approach included difficulty ascertaining the actual incidence of CHD, understanding the full phenotypic spectrum of a mutant gene, and the possibility of erroneously ascribing blame to a variant when other diagnoses are not considered. Furthermore, follow up functional studies to help validate pathogenicity were not always carried out. Several studies also used candidate gene approaches, testing cardiac developmental genes (mainly identified in mouse models) in human cohorts with similar CHD phenotypes. However, in some cases, at least, this produced misleading genotype–phenotype associations.

More recently, a different approach has been used. Over the last few years, three studies have employed whole exome sequencing (WES) in large cohorts of people with CHD to discover novel genes. Zaidi *et al*. ^8^ performed WES in 362 trios, in which the index had severe CHD, no known genetic diagnosis, and no family history of CHD. Results were compared to trios of parents and unaffected siblings from families with autism collected separately. Their analysis focussed on 4,169 genes with human orthologues that were in the top quartile for expression in an E14.5 mouse heart identified using RNA sequencing, called the high heart expression (HHE) gene group.

The authors found a significant excess of *de novo* protein-altering mutations in the HHE genes in individuals with CHD compared to controls. There was no significant difference between mutation load in genes seen in the lower quartile of heart expression and no increased burden in the CHD cohort compared to controls. Zaidi *et al.* identified eight mutations in the CHD cohort in genes that have a role in histone modification. Specifically, they were involved in production, removal, or reading of methylation of H3K4me (histone H3, lysine 4). This pathway already has links with CHD, as it includes *MLL2* (Kabuki syndrome), *KDM6A* (X-linked Kabuki syndrome), and *CHD7* (CHARGE syndrome). Other already established syndromes causing CHD result from abnormalities in histone modification, such as Rubinstein-Taybi syndrome ^9^. This H3K4me pathway was the only gene set that was significantly enriched in the CHD cohort, and there were no mutations in these genes in the control population.

Other genes of note included *WDR5*, *KDM5A*, *KDM5B*, *RNF20*, *UBE2B*, components of the H2BK120 ubiquitination complex, and *USP44*. *SMAD2* also demonstrated more mutations expected than by chance, as did *SUV420H1*, *MED20*, *HUWE1*, *CUL3*, *NUB1*, and *NAA15*. Both individuals with *SMAD2* mutations had dextrocardia, which is in keeping with a known involvement in left–right organisation ^10^. Overall, Zaidi *et al*. concluded that *de novo* protein-damaging mutations in hundreds of genes contributed to around 10% of severe CHD and that histone-modifying genes were significantly involved.

Following on from this initial study, Homsy *et al*. ^11^ examined 1,213 CHD trios without a recognisable genetic diagnosis, which included the 353 trios published by Zaidi *et al*. ^8^. They compared the number of *de novo* mutations in the CHD trios with 900 healthy trios. A significant enrichment (1.4-fold) of *de novo* predicted deleterious loss-of-function and missense variants was found in the CHD cohort as a whole. All phenotype groups except heterotaxy displayed this trend. In addition, there was a 2.4-fold increase in deleterious mutations in the top 25% of genes expressed during heart development, the HHE list created by Zaidi *et al*. ^8^. This was not seen in controls and supports the conclusions of Zaidi *et al.* that CHD often results from *de novo* mutations.

Homsy *et al.* also went on to consider the difference between syndromic and non-syndromic CHD (S-CHD and NS-CHD, respectively). This is an important distinction to make, as approximately 90% of people with CHD are non-syndromic and thus have isolated CHD. S-CHD is used to describe individuals with CHD and other congenital abnormalities and/or developmental delay. They identified significant enrichment of *de novo* predicted damaging mutations in S-CHD where both other non-cardiac congenital abnormalities and neurodevelopmental disability were present. This was highest in the HHE genes. They did not demonstrate any significant enrichment in the NS-CHD group, so the genetic mechanism underlying this phenotype remained unknown.

Homsy *et al*. estimated that *de novo* mutations in these HHE genes contributed to CHD in 20% of individuals with CHD, neurodevelopmental disorder, and congenital abnormalities, 10% of CHD with neurodevelopmental disorder, 6% of CHD with congenital abnormalities, and only 2% of NS-CHD. The authors suggested that reduced penetrance in the same genes could lead to both S-CHD and NS-CHD. They noted that a number of these genes are expressed in the developing heart and brain, suggesting a shared underlying genetic aetiology of CHD and neurodevelopmental disability.

To explore the possibility that certain genes appear to be important in the brain as well as in the heart, Homsy *et al*. went on to consider whether there was much overlap between genes with *de novo* damaging mutations in their CHD with neurodevelopmental disorders cohort, and 1,161 genes were identified with damaging *de novo* mutations in a population with neurodevelopmental disability without CHD. A total of 69 genes were shared between the two cohorts and were significantly enriched for *de novo* damaging mutations (2.6-fold enrichment). A number of these genes are important in cardiac development pathways, including the *WNT* and *NOTCH* pathways, and the group also included transcriptional regulators and chromatin modification genes. This led the authors to suggest that there is a common aetiology for both CHD and neurodevelopmental disorders with variable expressivity of these genes.

Homsy *et al.* also compiled a list of 21 genes with multiple damaging *de novo* mutations in the CHD cohort only, and not controls. These genes are likely to contribute to the pathogenesis of CHD, as this increased frequency of damaging mutations is unlikely to have occurred by chance. A number of genes already known to cause CHD were identified, such as *PTPN11* (Noonan syndrome), *KMT2D* ( *MLL2* Kabuki syndrome), *MYH6* (atrial septal defects, cardiomyopathy), *JAG1* (Alagille syndrome), *CHD7* (CHARGE syndrome), *ZEB2* (Mowat Wilson syndrome), and *NOTCH1* (aortic valve disease and Adams Oliver syndrome). *RBFOX2* was a novel gene. Three individuals from this study and an additional patient with a deletion of *RBFOX2* ^12^ had hypoplastic left heart syndrome. The authors suggest that, as this gene is needed for the correct development of the zebrafish heart ^13^ and is involved in epithelial-to-mesenchymal transformation, which is linked to HLHS ^14, 15^, it is likely to be a true CHD-causing gene. Genes with the Gene Ontology terms anatomic structure morphogenesis, cardiovascular system development, neurodevelopmental abnormality, and chromatin modification were also enriched in the CHD cohort. Chromatin modification was also highlighted in line with the results of Zaidi *et al.* ^8^, and these genes were still significant when the Zaidi cohort was removed from the analysis.

Thus, the genetic mechanisms behind the largest group with CHD, the NS-CHD group, remained unknown. We explored this as part of our recent worldwide collaboration with the Deciphering Developmental Disorders study and other researchers ^16^, which identified further novel CHD genes and uncovered a role for inherited mutations in NS-CHD. This is the largest CHD WES study to date and involved 1,891 probands with S-CHD and NS-CHD.

A significant excess of *de novo* protein-truncating variants (PTVs) and missense variants in genes already known to cause human autosomal dominant CHD in the S-CHD group was observed. The burden of *de novo* PTVs in these same genes was lower in the NS-CHD group, which supports the findings of Homsy *et al.* and the role of *de novo* mutations in S-CHD ^11^. A significant excess of *de novo* PTVs in the S-CHD cohort in genes known to cause developmental disability (DD) but not previously linked with CHD was also reported. This expands the phenotypes associated with these genes to include CHD and supports the theory that certain genes may be important in both the heart and the brain. The work also points to further novel genes to be discovered, as there was a significant exome-wide excess of *de novo* missense mutations in all other remaining protein-coding genes across both NS-CHD and S-CHD groups.

The role of inherited mutations was also considered. The burden of rare inherited variants between those with CHD and 12,031 population-matched controls was compared. In the NS-CHD group, there was a significant excess of rare inherited PTVs in genes known to cause autosomal dominant CHD. This excess was not present in the S-CHD group. This is a novel finding, suggesting a role for inherited mutations with reduced penetrance in NS-CHD. The variants were in genes known to cause NS-CHD or S-CHD where the phenotype can be mild and hard to detect ( *ABCC9, ACTC1, COL1A1, NOTCH1*, and *NOTCH2*, ** for example). This explains why mutations in genes known to cause S-CHD might be detected in a NS-CHD population. In addition, the *de novo* mutations in the S-CHD group and the inherited mutations in the NS-CHD group were in different genes with no overlap. This suggests that there are other novel genes that cause NS-CHD and exhibit incomplete penetrance still to be discovered because there was an exome-wide excess of rare inherited PTVs in the remaining protein-coding genes not known previously to be associated with CHD or DD. This was not seen in the S-CHD group.

Three novel S-CHD genes— *CDK13*, *CHD4*, and *PRKD1*—were also identified. Mutations in *CDK13* were clustered together in the serine/threonine protein kinase domain. Affected individuals have recognisable facial similarities. In addition, they all have septal defects and abnormalities of the pulmonary valve. It has been shown that the complex between *CDK13* and cyclin K phosphorylates RNA polymerase II and is needed for alternative RNA splicing ^17, 18^. *CHD4* is involved in chromatin remodelling as part of the NuRD complex. A mouse model has been produced without endothelial *Chd4*, which results in death at mid-gestation due to vascular rupture ^19^. There are similarities in the individuals’ facial features and phenotype, including developmental delay, genitourinary abnormalities, and structural brain abnormalities, which exhibits some overlap with CHARGE syndrome ( *CHD7*). Individuals with mutations in *PRKD1* exhibited CHD ** with developmental delay as well as ectodermal and limb features. *PRKD1* is a serine/threonine protein kinase that has been implicated in cardiac hypertrophy, and a conditional mouse model with a cardiac-specific *Prkd1* null allele results in reduced fibrosis and hypertrophy and improved cardiac function under pressure overload ^20^. A family with truncus arteriosus and recessive PTVs in *PRKD1* has already been reported in Saudi Arabia ^21^.

Genes related to chromatin modification, neural tube development, cardiac development, and protein phosphorylation were significantly over represented in the CHD cohort. A protein–protein interaction map for potentially significant CHD genes with connections greater than would be expected due to chance alone was produced (see Supplementary Figure 4 in Sifrim *et al.* ^16^), and this includes known CHD genes such as *NOTCH1*, *SOS1*, *SMAD4*, and *EP300*. Such interaction maps will help to focus future CHD functional studies.

While these studies represent exciting steps forward in our understanding of CHD genetics, there was little overlap of the novel genes identified and already known genes, emphasizing that there is a long way to go to discover all the genes involved in CHD. However, previous analyses restricted to specific CHD phenotypes (Tetralogy of Fallot) have demonstrated an overlap with genes with known cardiac roles in the sarcomere and neural crest, for example ^22^.

These studies already facilitate improvement to patient care. Multiple definitely pathogenic mutations in already established CHD genes have been identified, and these results are being fed back to the participants. Whilst a genetic diagnosis won’t necessarily alter the medical or surgical management of their heart defect, it may lead to recommendations of other medical checks or longer-term screening to identify other potential problems, including cardiomyopathy ^6^. It could also have implications for the wider family, who may be offered echocardiographic or genetic screening. Prenatal diagnosis with sonography, genetic testing, and the possibility of pre-implantation genetic diagnosis are all available, and couples may choose different options based on the risk of a foetus being affected. Hopefully, in the future, non-invasive prenatal diagnosis will also be possible. Parents often report that a genetic diagnosis is helpful, partly because having an explanation was beneficial and because many worried that they did something in the pregnancy to cause the heart problem.

These studies also help us learn about novel and already established genes. It appears that there is a recognisable phenotype for *CDK13* mutations that clinicians can look for when reviewing patients. The smaller numbers with *CHD4* and *PRKD1* mutations mean that the phenotype is yet to be established, although there are some consistent and distinct features, particularly for *CHD4* ^23^. Follow up of these individuals and identification of others who are affected will help us understand the phenotypic spectrum more fully. These studies have also expanded the phenotype of some well-known genes. For example, Zaidi *et al.* ^8^ reported an individual with a *CHD7* mutation with none of the significant features of CHARGE syndrome. Targeted testing based on an individual’s phenotype has created ascertainment bias in the past, and it is likely that the true range of phenotypes of even well-established CHD genes is not yet known. It is obvious that closer interaction between clinicians and researchers will be required in the future. An absence of mutations in genes with an established role in heart development, such as *NKX2*. *5*, was apparent in the data set examined by Sifrim *et al.* ^12^. This may reflect a truer incidence of the contribution of these genes to CHD rather than the previously published targeted sequencing studies. Even within the developmental disorders cohorts, there are mutations in genes such as *CDK13* and *CHD4* in individuals with and without CHD. Similarly, families with CHD often show non-penetrance or mutation carriers without a severe phenotype, highlighting the importance of detailed phenotyping but also pointing towards modifying factors. Thus, an approach focusing on severely affected individuals would allow the identification of currently unknown genes and open up future perspectives to study genotype–phenotype correlation in more detail in selected mutation carriers as well as mouse models.

Clearly, there are huge benefits to using WES and other non-targeted testing in large cohorts, but the possibility of incidental findings requires careful consideration. Variants detected in genes unrelated to the primary study objectives may have significant clinical consequences for the individual: for example, the detection of a *BRCA* gene mutation in a patient from a CHD cohort. There is some disagreement as to which incidental findings should be reported to the individuals concerned ^24, 25^ (see https://www.mrc.ac.uk/documents/pdf/mrc-wellcome-trust-framework-on-the-feedback-of-health-related-findings-in-researchpdf/ and https://www.genomicsengland.co.uk/wp-content/uploads/2015/03/GenomicEnglandProtocol_030315_v8.pdf). In addition, there are obvious difficulties interpreting variants of uncertain significance and other issues such as carrier status. These matters need to be carefully considered and included within the consent process.

We now know that much larger cohorts of individuals with CHD must be examined to discover the remaining CHD-causing genes. This will require collaboration between different research groups and extensive data sharing, which may provide challenges for data protection and participant confidentiality. Consent for genetic testing is already particularly complicated in terms of feedback, variant interpretation, and incidental findings, policies on which are still evolving but clearly need to be evidence-based. The privacy of a person’s data is obviously important, and the requirements to keep research data confidential have recently been highlighted by the PACE trial ^26^. Despite participants not consenting to release anonymised data, the courts forced data release in response to a freedom of information request because of a strong public interest. This was despite the researchers’ concerns that patients could potentially be identified. Open data and transparent research must be encouraged, but researchers might choose not to include data release as part of their consent process owing to concerns that it may put people off involvement in research. Funding agencies are playing an important role in driving data sharing, but more is needed from clinical diagnostic testing in addition to research cohorts.

The field of CHD genomics is rapidly changing, and there are many challenges facing researchers in this area. However, genomics research in CHD has the potential to immediately improve patient care by increasing the breadth of genetic testing for patients, clinical recognition of new syndromes, and tailored healthcare with better reproductive counselling and choices. This diverse population of individuals is increasing in numbers, and increased collaboration between researchers and clinicians is needed. Specifically, closer relationships among cardiologists, clinical geneticists, and researchers as well as genomic education for clinicians have been recommended ^27, 28^. Data sharing will be essential but must be done with careful consideration of participant confidentiality. Looking forwards, this is the dawn of a new era where the application of genomics can provide us with vital insights into the pathogenesis of disease and will help to transform patient care.

## Abbreviations

CHD, congenital heart disease; DD, developmental disability; HHE, high heart expression; NS-CHD, non-syndromic congenital heart disease; PTV, protein-truncating variant; S-CHD, syndromic congenital heart disease; WES, whole exome sequencing.
